# Gaucher Disease Diagnosis Using Lyso-Gb1 on Dry Blood Spot Samples: Time to Change the Paradigm?

**DOI:** 10.3390/ijms23031627

**Published:** 2022-01-30

**Authors:** Tama Dinur, Peter Bauer, Christian Beetz, Guido Kramp, Claudia Cozma, Marius-Ionuț Iurașcu, Michal Becker-Cohen, Majdolen Istaiti, Arndt Rolfs, Ari Zimran, Shoshana Revel-Vilk

**Affiliations:** 1 Gaucher Unit, Shaare Zedek Medical Center, Jerusalem 9103102, Israel; dinurtama@gmail.com (T.D.); michalbc@szmc.org.il (M.B.-C.); joleenist@szmc.org.il (M.I.); azimran@gmail.com (A.Z.); 2 Centogene GmbH, 18055 Rostock, Germany; Peter.Bauer@centogene.com (P.B.); christian.beetz@centogene.com (C.B.); guido.kramp@centogene.com (G.K.); claudia.cozma@centogene.com (C.C.); marius.iurascu@centogene.com (M.-I.I.); arndt.rolfs@arcensus-diagnostics.com (A.R.); 3 Medical Faculty, University of Rostock, 18051 Rostock, Germany;; 4 Arcensus GmbH, 18055 Rostock, Germany; 5 Faculty of Medicine, Hebrew University of Jerusalem, Jerusalem 9112002, Israel

**Keywords:** glucosylsphingosine, lyso-Gb1, Gaucher disease, dry blood spot, diagnosis

## Abstract

For years, the gold standard for diagnosing Gaucher disease (GD) has been detecting reduced β-glucocerebrosidase (GCase) activity in peripheral blood cells combined with *GBA1* mutation analysis. The use of dried blood spot (DBS) specimens offers many advantages, including easy collection, the need for a small amount of blood, and simpler transportation. However, DBS has limitations for measuring GCase activity. In this paper, we recount our cross-sectional study and publish seven years of experience using DBS samples and levels of the deacylated form of glucocerebroside, glucosylsphingosine (lyso-Gb1), for GD diagnosis. Of 444 screened subjects, 99 (22.3%) were diagnosed with GD at a median (range) age of 21 (1–78) years. Lyso-Gb levels for genetically confirmed GD patients vs. subjects negative to GD diagnosis were 252 (9–1340) ng/mL and 5.4 (1.5–16) ng/mL, respectively. Patients diagnosed with GD1 and mild *GBA1* variants had lower median (range) lyso-Gb1, 194 (9–1050), compared to GD1 and severe *GBA1* variants, 447 (38–1340) ng/mL, and neuronopathic GD, 325 (116–1270) ng/mL (*p* = 0.001). Subjects with heterozygous *GBA1* variants (carrier) had higher lyso-Gb1 levels, 5.8 (2.5–15.3) ng/mL, compared to wild-type *GBA1*, 4.9 (1.5–16), ng/mL (*p* = 0.001). Lyso-Gb1 levels, median (range), were 5 (2.7–10.7) in heterozygous *GBA1* carriers with Parkinson’s disease (PD), similar to lyso-Gb1 levels in subjects without PD. We call for a paradigm change for the diagnosis of GD based on lyso-Gb1 measurements and confirmatory *GBA1* mutation analyses in DBS. Lyso-Gb1 levels could not be used to differentiate between heterozygous *GBA1* carriers and wild type.

## 1. Introduction

Gaucher disease (GD), one of the most common lysosomal storage disorders, is caused by a mutant lysosomal enzyme β-glucocerebrosidase (GCase), leading to the storage of glucocerebroside (GC) and other glycolipids in various tissues; it also leads to a multi-system disease known for its great phenotypic heterogeneity [1,2]. The disease is characterized by hepatosplenomegaly, anemia, thrombocytopenia, and skeletal disease. GD has an estimated prevalence of 1:50,000–100,000 in the general population, but it has a predilection for Ashkenazi Jews, wherein 1:17 are carriers and about 1:800 are affected [3].

The current gold standard for diagnosing GD is the detection of reduced GCase activity in peripheral blood cells (traditionally compared to same-day normal controls), combined with mutation analysis at the DNA level of the glucocerebrosidase gene (*GBA1*), performed today by whole-gene sequence [1]. The great phenotypic heterogeneity of GD is explained, in part, by the many (>860) variants within the gene identified to date, as well as different genetic, epigenetic, and environmental factors [4]. Bone-marrow aspiration and biopsies for identifying Gaucher cells are no longer acceptable as a means to diagnose GD and should be performed only when another hematologic comorbidity is being evaluated [5].

Plasma biomarkers have been used over the years to diagnose and follow patients with GD [6,7,8]. Historical biomarkers (i.e., angiotensin-converting enzyme, ferritin, alkaline phosphatase, and high-density lipoprotein) are not specific to GD and, thus, could not be used for diagnosis [9,10,11,12,13,14]. The utility of more specific biomarkers, namely chitotriosidase and chemokine (C-C motif) ligand 18 (CCL18), is also limited, as they are not specific for GD [15,16], and one in 20 individuals is entirely deficient in chitotriosidase activity, owing to polymorphism in the *CHIT1* gene [17]. In a search for more specific and sensitive biomarkers, two groups independently identified the deacylated form of glucocerebroside, glucosylsphingosine (Lyso-Gb1), as a candidate biomarker. This has been confirmed to be almost ideal, with very high specificity and sensitivity for the diagnosis of GD [18,19].

Dried blood spot (DBS) specimens are collected by applying a few drops of blood onto printed circles on absorbent filter paper. The use of DBS for diagnosis offers many advantages, including easy collection; the need for only a small amount of blood; and simpler transportation, as samples can be shipped via regular mail, at room temperature. However, DBS has limited usefulness for the measurement of GCase activity [20]. Thus, other methods for easy and reliable screening for the diagnosis of GD are needed.

In this paper, we publish seven years of experience by using DBS samples for GD diagnosis. We believe that our data will help establish the use of lyso-Gb1 extracted from DBS samples as a simple and highly reliable method for GD screening.

## 2. Results

### 2.1. Study Cohort

In this cross-sectional study, 444 subjects were tested (117 (27%) children < 18 years). DBS samples were sent for *GBA1* molecular sequencing and lyso-Gb1 quantification. A third of the cases presented with clinical features, including splenomegaly, thrombocytopenia, short stature, etc. (Table 1). The rest were tested due to a family history of GD, as part of a research study in prodromal Parkinson’s disease (PD) and for the evaluation of *GBA1* mutation in patients recently diagnosed with PD.

The diagnosis rate of GD was 22.3% (99/444). About 50% of subjects evaluated for clinical features, and 25% of those evaluated for family history were diagnosed with GD (Table 1). The most common genotype was N370S (c.1226A > G) homozygous (Table 2a). Two patients evaluated for a recent diagnosis of PD were diagnosed with GD: one at the age of 55 years with N370S/R496H (c.1604G) variants and the second at the age of 52 years homozygous for the N370S variant. In addition, three asymptomatic subjects, all homozygous for the N370S variant, were screened as part of a prodromal PD study and were diagnosed with GD. 

**Figure 1:**
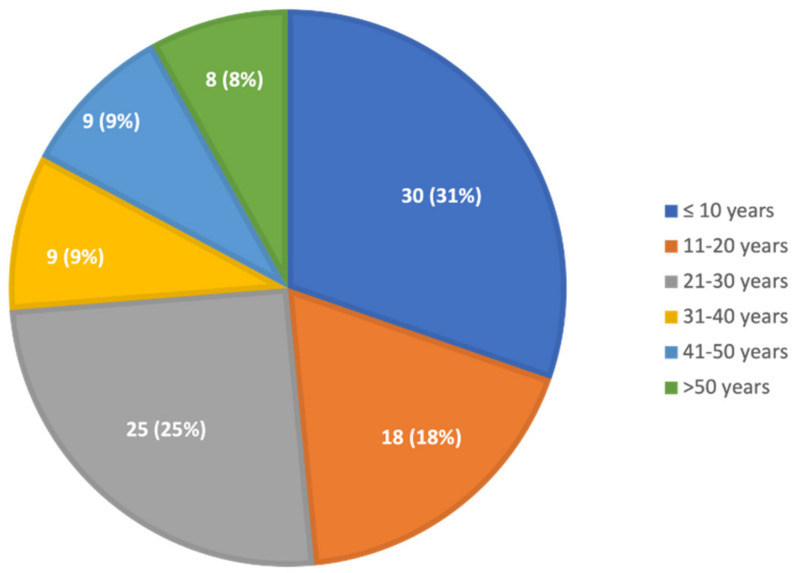
Age at diagnosis of GD. Thirty children were diagnosed at the age of 0–10 years, 18 at the age of 11–20 years, 25 at the age of 21–30 years, nine at the age of 31–40 years, nine at the age of 41–50 years, four at the age of 51–60 years, and four at the age of over 61 years.

The median age at diagnosis was 21 years; almost half were diagnosed when they were younger than 20 years, a quarter were 20–30, and a quarter were older than 30 years (Figure 1). The age at diagnosis was not different between males and females. The median (range) age at diagnosis was 21 (1–78) years for those with clinical symptoms and 16 (2–42) years for those evaluated for family history.

### 2.2. Lyso-Gb1 Levels for Diagnosis of Gaucher Disease

**Figure 2:**
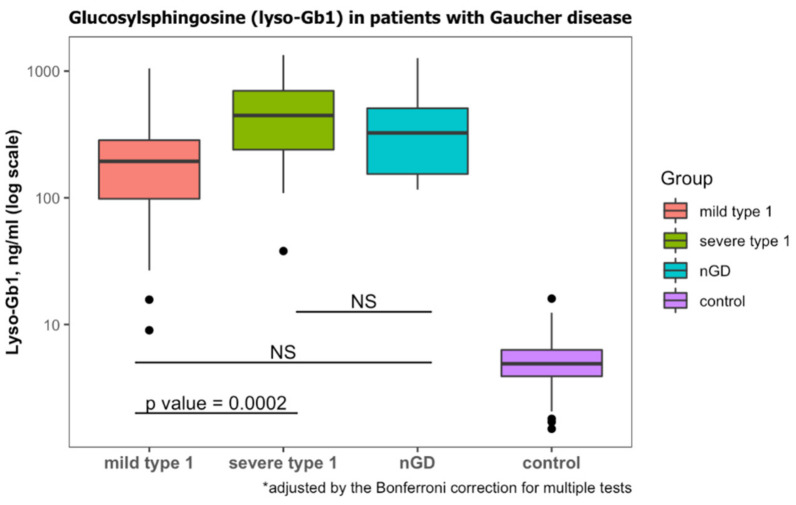
Lyso-Gb1 levels at diagnosis of GD. Classification of mild type 1, severe type 1 and neuronopathic GD (nGD) was based on genotypes. Lyso-Gb1 levels of subjects with wild-type *GBA1* were considered to be the control. NS, non-significant. Black dots represent the outliers.

According to laboratory protocol, lyso-Gb1 levels less than 6.8 ng/mL are considered normal. All patients diagnosed with GD had lyso-Gb1 levels that were higher than this threshold (Table 1). Those diagnosed with GD1 and mild *GBA1* variants had lower Lyso-Gb1 levels than those diagnosed with severe *GBA1* variants (Table 3, Figure 2). Lyso-Gb1 levels of the diagnosed with nGD were non-significantly different from mild GD1 and severe GD1 (Figure 2).

**Figure 3:**
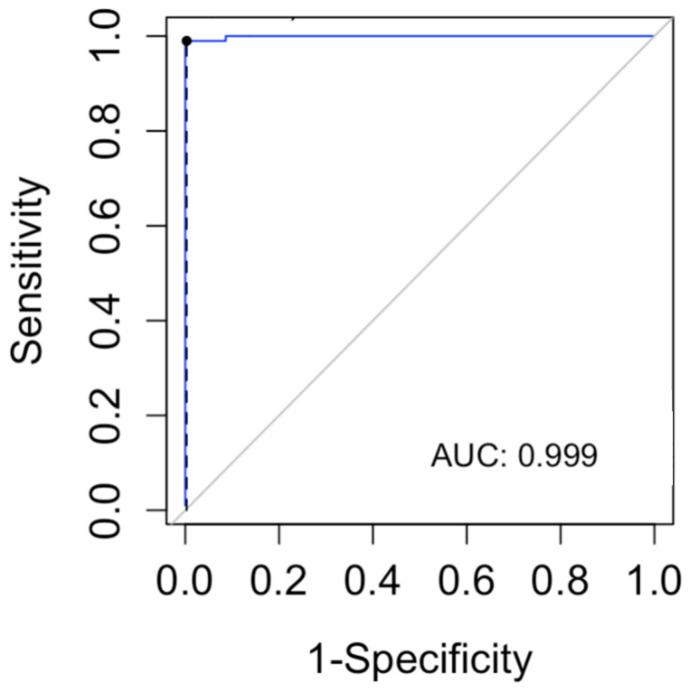
The receiver operating characteristic (ROC) curve was used to evaluate the lyso-Gb1 ability for classifying Gaucher disease vs. no Gaucher disease. The area under the curve (AUC) was calculated.

In order to determine a lyso-Gb1 cutoff level for the diagnosis of GD, we computed a ROC curve and searched for the cutoff with the maximum sensitivity for the diagnosis of GD (Figure 3). The cutoff found in our data was 9 ng/mL (sensitivity 100%, CI 96–100%). The specificity of this cutoff was 91.3% (95% CI 87.8–94.1%).

Lyso-Gb1 levels above 9 ng/mL without clinically relevant bi-allelic *GBA1* variants were more common in heterozygote *GBA1* carriers than in the wild type (*p* = 0.036) (Table S2). No association was found with age, sex, or cause of testing.

### 2.3. Lyso-Gb1 Levels for Diagnosis Heterozygous GBA1 Carrier against Wild Type

**Figure 4:**
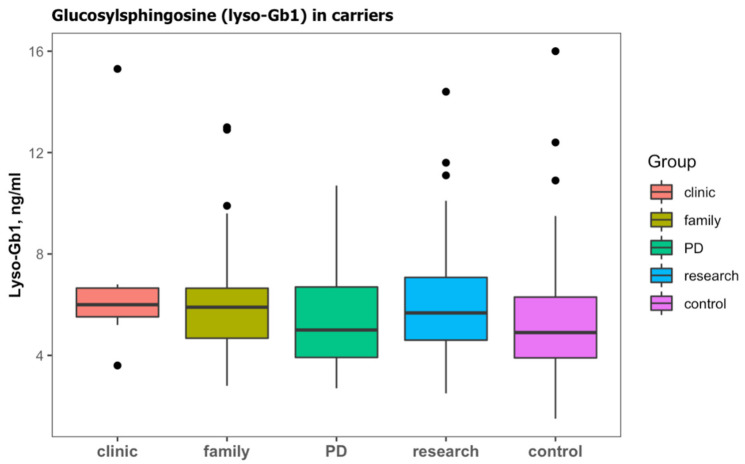
Lyso-Gb1 levels in subjects with monoallelic variants in *GBA1* (carriers) according to the cause for sending a sample; clinical features (clinic), family history of GD (family), Parkinson’s disease (PD), and subjects enrolled on a study of prodromal Parkinson’s disease (research). Lyso-Gb1 levels of subjects with wild-type *GBA1* were considered to be the control. Black dots represent the outliers.

In the cohort, 188 (42.3%) subjects were identified with a heterozygous *GBA1* carrier status (Table 2b). Those subjects were older than subjects with wild type and were mainly screened due to a family history of GD, a diagnosis of PD, or as part of research in prodromal PD (Table 4). Lyso-Gb1 was significantly higher in carriers than those with the wild type (Table 4). Carriers with PD had slightly lower lyso-Gb1, but this difference did not reach statistical difference (Figure 4).

**Figure 5:**
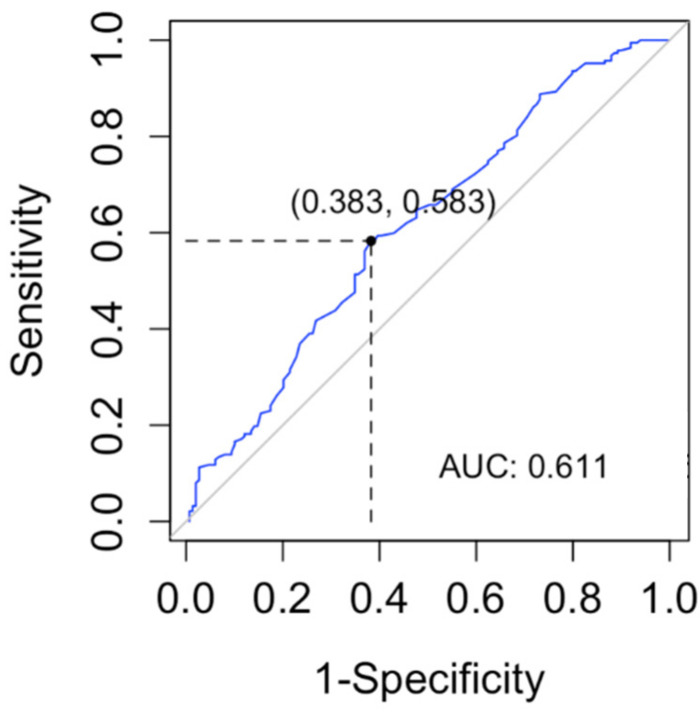
Receiver operating characteristic (ROC) curve was used to evaluate the lyso-Gb1 ability for classifying carrier vs. wild-type *GBA1*. The area under the curve (AUC) was calculated.

In order to determine a lyso-Gb1 cutoff level for the diagnosis of subjects with carrier status in *GBA1* vs. wild type, we computed a ROC curve and searched for the cutoff with the maximum sensitivity for the diagnosis of subjects with monoallelic variants in *GBA1* (Figure 5). The cutoff found in our data was 2.5 ng/mL (sensitivity 100%, CI 98–100%). The specificity of this cutoff was 6% (95% CI 2.7–11.1%).

## 3. Discussion

In our study, we confirmed the utility of DBS for diagnosing GD based on lyso-Gb1 levels and subsequent molecular analysis. All subjects with biallelic variants in *GBA1*, diagnosed as GD, had elevated lyso-Gb1, i.e., above 9 ng/mL. The combination of lyso-Gb1 and whole-gene sequence provided a 100% accurate diagnosis of GD, meaning that this can now become the new standard for screening of patients suspected to have GD. This recommendation updates the gold-standard diagnostic test (low activity of the glucocerebrosidase enzyme), which was defined by some of us in the two most recently published textbooks of hematology (see References [1,21]), as this current analysis had not been completed at the time those chapters were submitted. Severe cases of GD, all having significantly elevated lyso-Gb1 levels, would have been diagnosed definitively based on lyso-Gb1 testing alone. Lower lyso-Gb1 levels (less than 16 ng/mL) were also found in heterozygote GD carriers and individuals without relevant *GBA1* variants, using state-of-the-art exon sequencing and copy-number analysis. The levels of lyso-Gb1 could not be used to differentiate between the heterozygous *GBA1* carrier and wild type.

Early and accurate diagnosis of GD is essential to facilitate timely management decision-making, prevent unnecessary tests (some invasive), reduce anxiety induced by a lack of diagnosis, and importantly to prevent the birth of another sibling with potentially severe inherited disease [5]. Delayed diagnosis and misdiagnosis of GD, as for other rare diseases, remain major problems, due to nonspecific signs, symptoms, and laboratory findings; low awareness by healthcare professionals; and, in many cases, the lack of access to a simple diagnostic test. The inclusion of enzymatic GCase levels on fresh blood samples as the gold standard for the diagnosis of GD may be a major obstacle to the diagnosis of GD. Currently, very few laboratories are able to provide reliable enzymatic assays on blood samples. Most of them are research labs that cannot offer a clinical-scale activity or certification. In addition, the shipment of fresh blood samples is costly, and the number of samples sent at the same time is limited. The enzymatic GCase test needs a relatively large blood volume (6–10 mL) and preparation within 2 or 3 days. In contrast, the shipment of DBS cards is cheap, many cards can be sent at the same time, and the samples in the DBS remain stable for years. From a small volume of blood, both lyso-Gb1 levels and molecular analysis can be performed accurately.

The role of lyso-Gb1 in diagnosing GD is well established [19,22]. Our present study highlights the reliability of using lyso-Gb1 measured in DBS for diagnosing all types of GD in a large cohort of subjects who underwent testing for different reasons. Lyso-Gb1 measurement on DBS was also used in a case-control study conducted in the Russian Federation and was found to be highly sensitive and specific for GD [23]. In an Australian study, DBS measurements of lyso-Gb1 were also used for the prenatal diagnosis of GD [24]. The measurement of lyso-Gb1 levels from DBS has been used in newborn screening for GD [25].

In our study, we identified 99 new patients with GD. As expected, children with a severe genotype, i.e., nGD and compound heterozygote GD1, were diagnosed at an earlier age [26]. Disease severity based on genotype was also reflected by higher lyso-Gb1 levels. Since lyso-Gb1 was shown to have a role in the follow-up of treated and untreated patients [27,28,29,30], we believe that the inclusion of lyso-Gb1 in the diagnostic process can help in the assessment of the patient baseline status and for follow-up. For example, in an algorithm for asymptomatic patients who initially do not need treatment, an increase of Lyso-Gb1 levels could trigger the initiation of GD-specific therapy [2].

Subjects with monoallelic variants in *GBA1* have an increased risk of developing PD [31,32,33]. In a meta-analysis of patients with PD, it was found that carriers of the N370S mutation have a 3-fold higher risk for developing PD, while carriers of other *GBA1* mutations have up to a 15-fold higher risk for developing PD [34]. Although considerable clinical variation is seen [33], GBA-associated PD is typically associated with an earlier age of onset, a faster deterioration of motor functions, and a higher frequency and faster progression of cognitive decline compared to non GBA-associated PD, thus highlighting the importance of detection of the *GBA1* variant in patients with PD (as well as with other synucleinopathies; see Reference [32]). Although all subjects with heterozygous *GBA1* carrier status had a lyso-Gb1 above 2.5 ng/mL, the specificity of this cutoff was too low to recommend using lyso-Gb1 for screening for a heterozygous *GBA1* variant. Lyso-Gb1 levels in subjects with PD were similar to those screened for other reasons. Plasma lyso-Gb1 accumulation is, therefore, unlikely to be related to the higher risk of PD in monoallelic *GBA1* variants [35,36].

## 4. Materials and Methods

**Figure 6:**
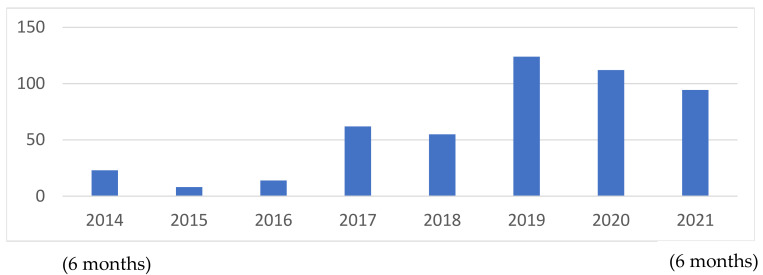
Number of patients tested per year over the seven years of study.

All DBS samples sent for *GBA1* molecular sequencing and lyso-Gb1 from the Gaucher Unit, Shaare Zedek Medical Center, from July 2014 to July 2021, were included in the study. The number of tests performed increased with time (Figure 6).

All samples were analyzed in Centogene GmbH, Rostock, Germany. Genotyping was performed by whole *GBA1* sequencing (exons and exon-intron boundaries) from DNA extracted from all DBS samples independent of the lyso-Gb1 levels. The sequencing approach would have detected all types of *GBA*1 aberrations, including GBAP-mediated recombination events. The definitions of mild versus severe genotypes were determined by N370S (c.1226A > G) homozygous and N370S/R496H (c.1604G) compound heterozygous were categorized as “mild”, whereas all other genotypes as “severe”. Lyso-Gb1 levels were performed according to the previously described method [19].

### 4.1. Biological Material

The material used was DBSs prepared by dropping 60 µL blood on CentoCard^®^ filter paper (Centogene GmbH, Rostock, Germany); the spots were allowed to dry for 2–4 h at room temperature. The filter cards are the ideal material for shipping and long-term storage. Before analysis, 3.2 mm discs were cut from the homogeneous parts of the DBS, using a DBS puncher (Perkin Elmer LAS GmbH, Hamburg, Germany). Each disc contains approximately 3.1 µL blood.

### 4.2. Sample Preparation

For each subject, 3 DBS discs were cut and collected into a round-bottom 2 mL tube (Sarstedt AG & Co. KG, Nümbrecht, Germany). Then 50 µL extraction solution (DMSO/water, 1/1) and 100 µL internal standard solution (200 ng/mL Lyso-Gb2 (Biotrend Chemikalien GmbH, Köln, Germany) in ethanol, were added to the tube and incubated for 30 min, at 37 °C, under agitation at 700 rpm. The samples were briefly sonicated (1 min) before they were transferred to a PALL-8048 96-well filter plate with a PTFE membrane (WVR International GmbH, Dresden, Germany) on top of a 96-well V-shaped plate (WVR International GmbH, Dresden, Germany). The cellular and paper debris were filtered by centrifugation for 5 min, at 3500 rpm, in a Hermle Z300 plate centrifuge (Hermle Labortehnik GmbH, Wehingen, Germany). The V-shaped plate was covered with aluminum foil and inserted into the sample manager.

### 4.3. LC/MS Method

The samples were separated by liquid chromatography on an ACE 3 C8, 50 × 2.1 mm column (MZ-Analysentechnik GmbH, Mainz, Germany), using a Waters I-Class UPLC (Waters GmbH, Eschborn, Germany). Solvents: 50 mM FA in water (A) and 50 mM FA in acetone/acetonitrile 1/1 (B). A flow rate of 0.9 mL/min preheated at 60 °C was used. The gradient was linear, and the analytes were eluted between 40% and 70% B solvent.

The UPLC was coupled with an AB-Sciex TQ-5500 (AB Sciex Germany GmbH, Darmstadt, Germany) mass spectrometer, using a 3:1 flow splitter. An MRM method was used for monitoring the analytes with the following settings: curtain gas, 40 psi; ion spray, 5500 V; desolvation temperature, 500 °C; declustering potential, 40 V; entrance potential, 10 V; collision energy, 30 V; and MRM transitions, Lyso-Gb1 (462.3–282.2) and Lyso-Gb2 internal standard (624.3–282.2).

### 4.4. Quantification

A 7-points calibration line was added to each plate before measurement. The preparation was similar to the samples, but the DBSs were replaced with standard solutions of increasing Lyso-Gb1 (Biotrend Chemikalien GmbH, Köln, Germany) concentrations: 0, 1, 5, 10, 50, 100, and 200 ng/mL. Analysis and quantification were performed by using the Analyst 1.6.2 software (AB Sciex Germany GmbH, Darmstadt, Germany).

### 4.5. Statistical Analysis

To report summary descriptive statistics, we used median (range) for continuous variables. For nominal data, we report the absolute and relative frequencies. The Kruskal–Wallis H test was used to determine statistically significant differences between two or more groups of an independent variable on a continuous or ordinal dependent variable. A chi-square test was used to compare categorical data. The optimal.cutpoints r program with maximum sensitivity analysis was used to calculate the optimal cut point for diagnosing GD and monoallelic *GBA1* variant and plotting the ROC curves [37]. The criterion was based on the maximization of sensitivity [38,39,40]. If more than one cut point fulfills this condition, those that yield maximum specificity are chosen. A two-sided significance level of α = 0.05 was considered.

## 5. Conclusions

Based on our results and others, we call for a shift in the diagnostic approach to GD based on lyso-Gb1 measured in DBS accompanied with molecular analysis of *GBA1*, without the need to confirm the diagnosis with blood-based measurement of GCase enzyme activity.

## Figures and Tables

**Table 1:** Characteristics of subjects with and without a diagnosis of Gaucher disease.

		Gaucher Disease		
	Total	Yes	No	*p*
n	444	99	345	
Male, n (%)	202 (46%)	41 (42%)	161 (47%)	NS
Age, years: median (range)	34 (1–90)	21 (1–78)	42 (1–90)	<0.001
Reason for screening				<0.001
Clinical features, n (%)	138 (31.1%)	67 (67.7%)	71 (20.6%)	
Family study, n (%)	156 (35.1%)	27 (27.3%)	129 (37.4%)	
Parkinson’s disease, n (%)	20 (4.5%)	2 (2%)	18 (5.2%)	
Research, n (%)	130 (29.3%)	3 (3%)	127 (36.8%)	
Lyso-Gb1, ng/mL: median (range)	6.1 (1.5–1340)	252 (9–1340)	5.4 (1.5–16)	<0.001

NS, non-significant; Research, subjects enrolled on a study of prodromal Parkinson’s disease.

**Table 2:** Distribution of genotypes in subjects with or without the diagnosis of Gaucher disease.

(a) Subjects Diagnosed with GD (n = 99), n (%)		(b) Heterozygote GBA1 Carriers (n = 188), n (%)	
N370S/N370S	58 (58.6)	N370S/wt	126 (67)
N370S/84GG	8 (8.1)	84GG/wt	23 (12.2)
N370S/V394L	6 (6.1)	L444P/wt	18 (9.6)
L444P/L444P	5 (5.1)	R496H/wt	7 (1.6)
D409H/D409H	3 (3)	V394L/wt	5 (2.7)
N370S/L444P	3 (3)	D409H/wt	2 (1.1)
N370S/RecNci	3 (3)	247C > T/wt	1 (0.5)
R48W/L444P	3 (3)	85T/wt	1 (0.5)
N370S/IVS2 + 1	2 (2)	IVS/wt	1 (0.5)
84GG/R496H	1 (1)	p330*/wt	1 (0.5)
N370S/IVS	1 (1)	R48W/wt	1 (0.5)
N370S/M123T	1 (1)	W184R/wt	1 (0.5)
N370S/R496H	1 (1)	V394L/wt	1 (0.5)
N370S/RecNCi, del55	1 (1)		
Pr463c/c960-4del	1 (1)		
R48W/R48W	1 (1)		
V394L/84GG	1 (1)		

GD, Gaucher disease. Variants are based on the original allele descriptions. Conversion to the new cDNA and protein nomenclatures is available in Table S1.

**Table 3:** Genetic variants in patients with Gaucher disease.

	Type 1 Mild *	Type 1 Severe *	nGD **	*p*
n	59	30	9	
Male, n (%)	26 (41%)	14 (46.5%)	3 (33.3%)	NS
Age, years: median (range)	25 (2–78)	11 (2–35)	2 (1–11)	<0.001
Referral cause				NS
Clinical features, n (%)	37 (63%)	22 (71%)	8 (90%)	
Family study, n (%)	17 (29%)	9 (29%)	1 (10%)	
Parkinson’s disease, n (%)	2 (3%)	0	0	
Research, n (%)	3 (5%)	0	0	
Lyso-Gb1, ng/mL: median (range)	194 (9–1050)	447 (38–1340)	325 (116–1270)	0.001

nGD, neuronopathic Gaucher disease; NS, non-significant; Research, subjects enrolled on a study of prodromal Parkinson’s disease. * N370S (c.1226A > G) homozygous and N370S/R496H (c.1604G) compound heterozygous were categorized as “Type 1 Mild”, whereas all other genotypes were categorized as “Type 1 Severe”. ** Genotypes in nGD included L444P (c.1448T > C) homozygous (n = 5) and D409H (c. 1342G > C) homozygous (n = 3) and one patient with V394L/84GG (c.1297G/c.84dupG).

**Table 4:** Characteristics of carriers vs. those with wild type *GBA1*.

	Heterozygous Carrier	Wild Type	*p*
Number	188	157	
Male, n (%)	82 (44%)	79 (50.6%)	NS
Age, years: median (range)	49 (1–90)	25 (1–80)	<0.001
Referral cause			<0.001
Clinical features, n (%)	8 (4.3%)	65 (41.4%)	
Family study, n (%)	61 (32.4%)	68 (42.7%)	
Parkinson’s disease	17 (9%)	1 (0.6%)	
Research, n (%)	102 (54.3%)	25 (15.9%)	
Lyso-Gb1, ng/mL: median (range)	5.8 (2.5–15.3)	4.9 (1.5–16)	0.001

Research = subjects enrolled on a study of prodromal Parkinson’s disease.

## Data Availability

Data cannot be shared due to ethical and privacy issues.

## Supplementary Materials

The following supporting information can be downloaded at https://www.mdpi.com/article/10.3390/ijms23031627/s1.
