# Supplementary material for: Gaucher Disease Diagnosis Using Lyso-Gb1 on Dry Blood Spot Samples: Time to Change the Paradigm?
Source: Int J Mol Sci. 2022 Jan 30;23(3):1627. doi: 10.3390/ijms23031627 (PMC8835963; doi:10.3390/ijms23031627)
Supplement: Supplementary file 1 [file ijms-23-01627-s001.zip › Table S1.pdf]

Table S1. Conversion table for variants appearing in the manuscript to the proper cDNA and protein nomenclatures.

Original Allele Description	Proper cDNA Nomenclature	Proper Protein Nomenclature
247C>T	c.247C>T	p.R83C
84GG	c.84dup	p.L29Afs*18
85T	c.371T>C	p.M124T
D409H	c.1342G>C	p.D448H
c960-4del	c.960_964del	p.D321Pfs*19
del55	c.1265_1319del	p.L422Pfs*4
IVS, IVS2+1	c.115+1G>A	p.?
L444P	c.1448T>C	p.L483P
M123T	c.485T>C	p.M162T
N370S	c.1226A>G	p.N409S
p330*	c.990G>A	p.W330*
Pr463c	c.1504C>T	p.R502C
R48W	c.259C>T	p.R87W
R496H	c.1604G>A	p.R535H
RecNci	c.[1448T>C; c.1483G>C; 1497G>C]	p.[L483P; p.A495P; p.V499V]
V394L	c.1297G>T	p.V433L
W184R	c.667T>C	p.W223R
