# Supplementary material for: Gaucher Disease Diagnosis Using Lyso-Gb1 on Dry Blood Spot Samples: Time to Change the Paradigm?
Source: Int J Mol Sci. 2022 Jan 30;23(3):1627. doi: 10.3390/ijms23031627 (PMC8835963; doi:10.3390/ijms23031627)
Supplement: Supplementary file 1 [file ijms-23-01627-s001.zip › Table S2.pdf]

Table S2. Subjects not diagnosed with Gaucher disease and lyso-Gb1 levels above 9 ng/mL.

	Genotype	YOT	Age, Years	Sex	Reason for Testing	Lyso-Gb1, ng/mL
**1**	wt/wt	2019	2	F	Clinic	16
**2**	N370S/wt	2019	60	F	Clinic	15.3
**3**	N370S/wt	2019	42	M	Research	14.4
**4**	N370S/wt	2020	35	M	Family	13
**5**	N370S/wt	2021	7	M	Family	12.9
**6**	wt/wt	2021	42	F	Family	12.4
**7**	N370S/wt	2021	54	M	Research	11.6
**8**	N370S/wt	2020	46	F	Research	11.1
**9**	wt/wt	2021	60	M	Clinic	10.9
**10**	R496H/wt	2020	43	M	Parkinson's	10.7
**11**	N370S/wt	2020	73	F	Parkinson's	10.4
**12**	N370S/wt	2021	71	F	Research	10.1
**13**	N370S/wt	2020	31	F	Family	9.9
**14**	N370S/wt	2020	50	M	Research	9.8
**15**	N370S/wt	2020	74	F	Parkinson's	9.7
**16**	84GG/wt	2014	79	M	Research	9.6
**17**	L444P/wt	2014	79	F	Research	9.6
**18**	L444P/wt	2021	31	F	Family	9.6
**19**	N370S/wt	2021	61	F	Family	9.5
**20**	wt/wt	2020	-	F	Family	9.5
**21**	N370S/wt	2021	70	M	Research	9.3
**22**	N370S/wt	2020	40	M	Family	9.2
**23**	N370S/wt	2020	48	F	Research	9.2
**24**	N370S/wt	2017	42	F	Research	9.2
**25**	L444P/wt	2020	14	F	Family	9.2
**26**	N370S/wt	2018	72	M	Research	9.1
**27**	wt/wt	2020	2	M	Family	9.1
**28**	wt/wt	2020	8	F	Clinic	9.1
**29**	wt/wt	2018	28	F	Family	9.1

YOT, year of testing; wt, wild type; F, female; M, male; Research, subjects enrolled on a study of prodromal Parkinson's disease. Variants are based on the original allele descriptions. Conversion to the new cDNA and protein nomenclatures is available in Table S1.
